# The Skin Microbiome of Cohabiting Couples

**DOI:** 10.1128/mSystems.00043-17

**Published:** 2017-07-20

**Authors:** Ashley A. Ross, Andrew C. Doxey, Josh D. Neufeld

**Affiliations:** Department of Biology, University of Waterloo, Waterloo, Ontario, Canada; Oregon State University

**Keywords:** cohabitation, high-throughput sequencing, human skin, microbiome, random forest modeling

## Abstract

Distinct microbial communities inhabit individuals as part of the human skin microbiome and are continually shed to the surrounding environment. Microbial communities from 17 skin sites of 10 sexually active cohabiting couples (20 individuals) were sampled to test whether cohabitation impacts an individual’s skin microbiome, leading to shared skin microbiota among partner pairs. Amplified 16S rRNA genes of bacteria and archaea from a total of 340 skin swabs were analyzed by high-throughput sequencing, and the results demonstrated that cohabitation was significantly associated with microbial community composition, although this association was greatly exceeded by characteristics of body location and individuality. Random forest modeling demonstrated that the partners could be predicted 86% of the time (*P* < 0.001) based on their skin microbiome profiles, which was always greater than combinations of incorrectly matched partners. Cohabiting couples had the most similar overall microbial skin communities on their feet, according to Bray-Curtis distances. In contrast, thigh microbial communities were strongly associated with biological sex rather than cohabiting partner. Additional factors that were associated with the skin microbiome of specific body locations included the use of skin care products, pet ownership, allergies, and alcohol consumption. These baseline data identified links between the skin microbiome and daily interactions among cohabiting individuals, adding to known factors that shape the human microbiome and, by extension, its relation to human health.

**IMPORTANCE** Our work characterizes the influence of cohabitation as a factor influencing the composition of the skin microbiome. Although the body site and sampled individual were stronger influences than other factors collected as metadata in this study, we show that modeling of detected microbial taxa can help with correct identifications of cohabiting partners based on skin microbiome profiles using machine learning approaches. These results show that a cohabiting partner can significantly influence our microbiota. Follow-up studies will be important for investigating the implications of shared microbiota on dermatological health and the contributions of cohabiting parents to the microbiome profiles of their infants.

## Abstract (precis)

Our work characterizes the influence of cohabitation as a factor influencing the composition of the skin microbiome. Although the body site and sampled individual were stronger influences than other factors collected as metadata in this study, we show that modeling of detected microbial taxa can help with correct identifications of cohabiting partners based on skin microbiome profiles using machine learning approaches. These results show that a cohabiting partner can significantly influence our microbiota. Follow-up studies will be important for investigating the implications of shared microbiome on dermatological health and the shared contributions of cohabiting parents to the microbiome profiles of their infants.

## INTRODUCTION

Skin is the largest organ of the body, forming a critical protective barrier between an organism and its environment. The average human is covered by 1.5 to 2.0 m^2^ of skin, varying from 2 to 3 mm in depth (1). Skin is divided into three tissue layers: epidermis, dermis, and hypodermis. The epidermis is of particular interest because it is the exposed layer that contains a diverse microbial community of largely beneficial and benign microorganisms (2) while protecting the body from transient microorganisms with the potential to cause disease. This outer layer is relatively hostile to microbes because it is constantly shedding, associated with antimicrobial compounds, and low in moisture, yet with high acidity, hydrophobicity, and salinity (3). Despite these relatively harsh conditions, between one million and one billion microorganisms inhabit each square centimeter of skin (4, 5).

Microbial surveys demonstrate that human skin is inhabited by a diverse community of bacteria, archaea, fungi, protozoans, and arthropods (4, 6–8). In general, four bacterial phyla dominate the skin: *Proteobacteria*, *Actinobacteria*, *Firmicutes*, and *Bacteroidetes* (2, 9). In one survey of skin microbiota, *Propionibacteria*, *Corynebacteria*, and *Staphylococcus* spp. comprised more than 62% of sequences detected at 20 body sites (10). Moist, oily, and dry regions were each associated with different levels of microbial species diversity, which was lowest in oily regions that were associated with the sebaceous glands (10).

Human skin microbiota is strongly defined by body region and individuality (7). The main factors affecting the skin microbiota are skin location, biological sex, geographical location, ethnicity, skin depth, antibiotics, cosmetics, age, and health (11). Hygiene practices also influence the skin microbiome, including the use of lotions, antibiotics, soaps, and cosmetics (12, 13). Three-dimensional (3D) molecular cartography maps of human skin demonstrated that the molecular composition of human skin is defined by the microorganisms present, molecules from hygiene products, and local skin anatomical structure (1).

Although cohabitation may impact an individual’s skin microbiome, no study has yet explored the relationship between the skin microbial communities from a wide range of skin locations of intimate cohabiting couples. A previous human study determined that the palms of hands, oral cavity, and gut microbiomes were more similar within families (14). Moreover, humans and animals share a microbial community that is affected even by a short absence of several days (15). A study on the effects of antibiotic use on cohabiting individuals found household-specific microbial communities, although there was no significant difference between the number of shared taxa (16). To the best of our knowledge, there are no other studies on cohabitation that determined which skin regions are the most related between partners.

The objectives of this investigation were to characterize the distribution of bacteria and archaea on the skin of intimate cohabiting heterosexual couples and determine whether cohabitation leads to a detectable impact on the microbiome of partnered individuals compared to the influences of factors related to individuality and lifestyle. Although we confirm that the two dominant factors that influence the skin microbiome are individuality and body location, cohabitation was significantly associated with microbial community composition. Using random forest modeling, we found that the skin microbiome profiles of cohabiting couples can be correctly matched with significantly higher probability than by chance alone, especially based on data from foot swab samples. Thus, our results show that several factors are stronger than cohabitation in their associations with human skin microbiome variability, yet the imprint of one individual on a cohabiting partner can nonetheless be detected by microbiome analysis and machine learning approaches.

## RESULTS AND DISCUSSION

### Moisture level and individuality strongly influence microbial diversity.

**FIG 1:**
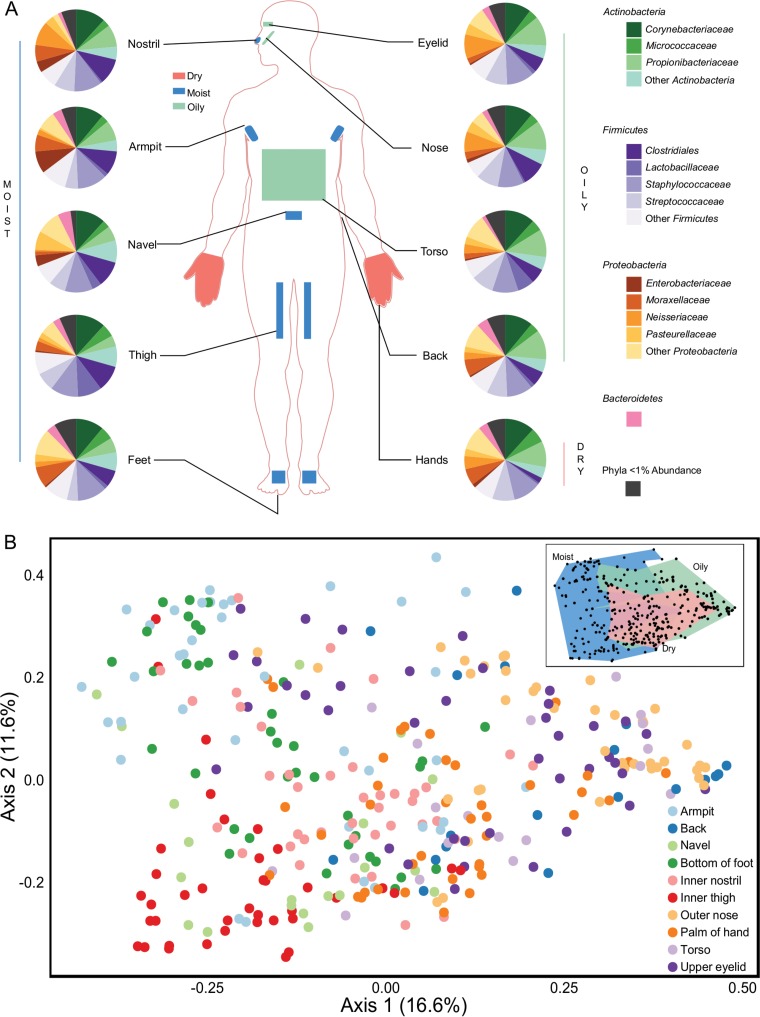
Microbial diversity of the 10 body locations sampled. (A) Pie charts illustrating the relative abundance of microbial families present at >1% and the phyla to which they belong, organized by each of the 10 body locations sampled. (B) PCoA plot calculated using the Bray-Curtis dissimilarity metric. The 330 samples from all body locations are included and are denoted by body location.

We analyzed the diversity of bacteria and archaea on 330 skin samples obtained from 17 skin regions of 10 sexually active cohabiting couples, which yielded 8,753,153 reads. A total of 4,639 unique operational taxonomic units (OTUs) were obtained from 1,746,690 sequences, rarefied to 5,293 reads per sample. Five no-template PCR controls and the sterile swab sample all contained fewer reads than the rarefied sequence count and produced no visible bands on an agarose gel following PCR amplification. Across all samples, the most abundant phyla were *Actinobacteria*, *Firmicutes*, and *Proteobacteria*, which is consistent with findings from previous human skin microbiome studies (2, 5, 9). These three phyla constituted 94.9% ± 5.2% of all reads (Fig. 1A), whereas the remaining sequences were affiliated with 38 phyla. *Bacteroidetes* was the only other phylum present above a relative abundance of 1%. *Archaea* comprised only 66 of the 16S rRNA gene sequences (0.004%), yet this may be an underrepresentation of their actual abundance because of known primer mismatches to archaeal 16S rRNA genes (17). The most abundant OTU was the common skin bacterium *Staphylococcus epidermidis* (18), which constituted 14.5% ± 15.4% of all sequences. There were 11 OTUs that were present above 1% relative abundance across the complete data set, representing 54.3% ± 23.1% of all sequences.

Known moisture levels of each body location were strongly associated with the observed variation in skin microbial communities (Fig. 1B). Grouping of samples from these three moisture levels was statistically significant (*F*_2,327 _= 18.8 by permutational analysis of variance [PERMANOVA]; *P* < 0.001). Significant variations in the abundance of *Staphylococcus*, *Corynebacterium*, and *Propionibacterium* were observed among moisture levels (Fig. 1A), as were alpha diversity differences (see Fig. S1 and Table S1 in the supplemental material). Similar shifts in abundance, such as high levels of *Propionibacterium* on facial skin sites, have been noted previously (6, 10). The Shannon index demonstrated that dry palm samples were significantly more diverse (*P* = 0.003 by nonparametric two-sample *t* test) than samples from both moist and oily regions, which had similar average Shannon diversity values. Palms and feet were the most diverse sites sampled and had significantly higher numbers of OTUs, likely due to their direct and frequent contact with microbiota from the environment.

10.1128/mSystems.00043-17.1 FIG S1  Boxplots of diversity by 10 body locations. Both the Shannon index and number of OTUs were plotted for all 330 samples. Download FIG S1, PDF file, 0.05 MB. Copyright © 2017 Ross et al. 2017 Ross et al. This content is distributed under the terms of the Creative Commons Attribution 4.0 International license.

10.1128/mSystems.00043-17.6 TABLE S1  Alpha diversity table containing Shannon index and OTU count averages, standard deviations, *t* statistics, and *P* values. The table contains multiple tabs. The first tab (leftmost tab) contains the Shannon index results for all samples, while the second tab contains the OTU counts for all samples. The third and fourth tabs are organized by each of the 10 body locations for Shannon index and OTU counts, respectively. Only metadata categories that were statistically significant were included. The fifth tab contains the summary of closest matches for each body location according to Bray-Curtis distance and weighted and unweighted UniFrac distances. Download TABLE S1, XLSX file, 0.1 MB. Copyright © 2017 Ross et al. 2017 Ross et al. This content is distributed under the terms of the Creative Commons Attribution 4.0 International license.

**FIG 2:**
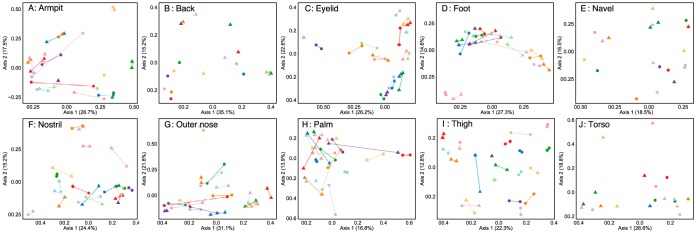
Ordinations (PCoA) generated by using the Bray-Curtis dissimilarity metric for each of the 10 body locations sampled. Lines connect samples from a participant. Female samples are denoted by circular points, whereas male partners are represented by triangles. Where a single sample per person was collected for specific body locations (i.e., back, navel, torso), no lines connect the participant samples. Samples from different couples are indicated by the different colors.

**FIG 3:**
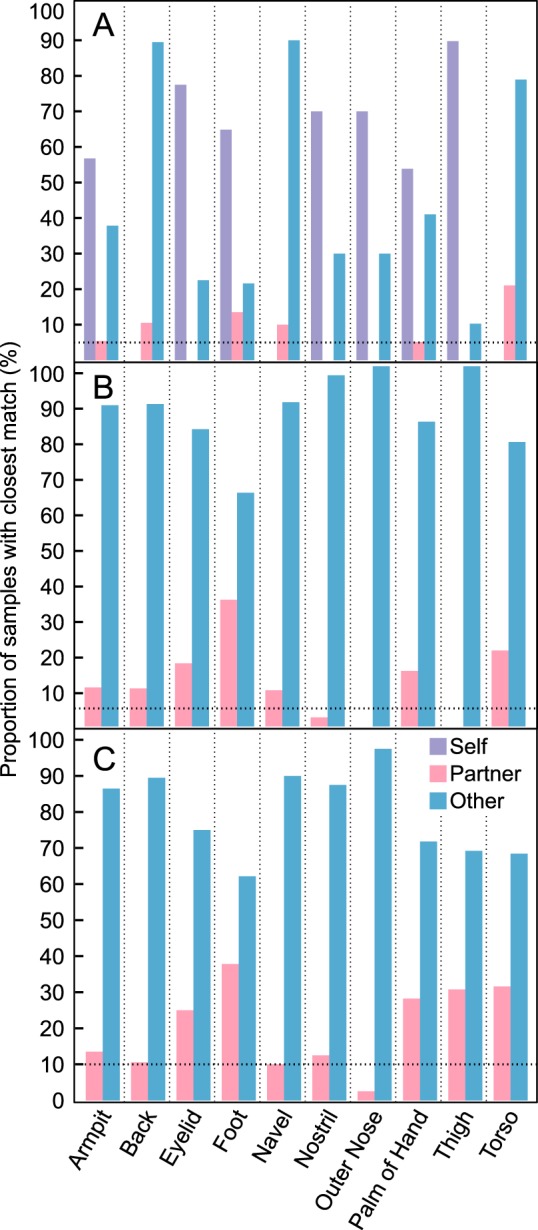
Samples were matched with another sample in the data set that possessed the most similar microbial community. Matches were analyzed to determine the percentage of samples belonging to self, partner, or another participant. (A) Proportion of samples that had the lowest Bray-Curtis distance with either another sample from within an individual, from within a cohabiting couple, or to any of the other participants. (B) Proportion of samples that had the lowest Bray-Curtis distance with nonself samples. (C) Proportion of samples that had the lowest Bray-Curtis distance with nonself, opposite-sex samples. The dotted line represents the threshold that would be expected by random chance from the 20 participants.

**FIG 4:**
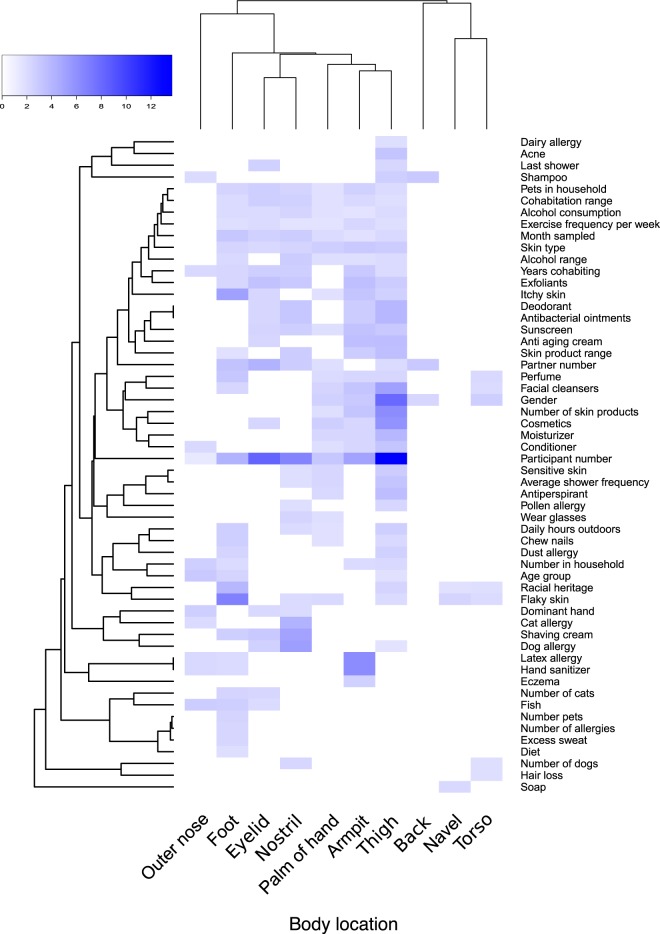
Heatmap summarizing the significant (*P* < 0.05) metadata factors that were collected from a participant survey. Categories with higher *F* values by PERMANOVA have higher variation in community dissimilarity within 10 body locations. White regions of the heatmap represent nonsignificant results. Body locations and metadata categories were arranged into dendrograms using the Bray-Curtis dissimilarity metric. Back, navel, and torso sites were associated with single samples, which likely contributed to fewer significant *F* values compared to other locations that were sampled on each side of the body.

Strong individuality was reflected by 16S rRNA gene profiles and supports previous research that suggested that individuality shapes the skin microbiome (7). When each body location was analyzed separately, the left and right replicates of each body location from an individual were associated with highly similar microbial profiles in the majority of cases (Fig. 2). When comparing Bray-Curtis distances, a sample had the lowest distance to another sample from the same participant 57.9% of the time. Within-individual closest matches (“individuality”) were highest for the thigh (89.7%) and eyelid (77.5%) sites (Fig. 3A) and were statistically significant (*F*_19,310 _= 3.65 by PERMANOVA; *P* < 0.001) when all samples were analyzed. Furthermore, when focusing on individual body locations with a minimum of two samples per participant, the *F* ratio increased (*F*_19,20 _= 3.12 to 13.69 by PERMANOVA; *P* < 0.001) and “Participant number” was the metadata category that explained the most variation for each body location, except for the outer nose (Fig. 4). The weakest individuality and strongest individuality were exhibited by the outer nose and thigh, respectively. We hypothesize that the thigh exhibited high individuality because it is a skin region that is not frequently in contact with the external environment, compared to other regions such as the hands. Furthermore, the left and right thighs are in more frequent contact with each other than other body regions, such as the left and right outer nose. Areas of the face with stronger individuality, such as the eyelids, may experience fewer perturbations from hand contact than the nose, especially for participants who wore glasses. Participants who did not wear glasses had modestly more eyelid microbial community similarity to their hands than those with glasses, according to Bray-Curtis distances (0.64 versus 0.59; *P* = 0.12); however, this was not statistically significant given the small sample size. We hypothesize that the outer nose may also express the lowest individuality due to the low level of diversity observed, which was lower than any other body region. Such low diversity may impact the ability to discern an individual due to fewer OTUs that are unique. Furthermore, a previous study of the face microbiome determined that microbial communities of this body region are strongly influenced by measured sebum and moisture levels in specific locations (19), which could result in similar communities between different individuals with similar secretion levels, which were not measured in this current study.

### Biological sex can be determined from thigh skin microbiome samples.

Female skin microbial communities were significantly more diverse than males, according to the Shannon index (4.59 versus 3.98; *P* < 0.001; Table S1), which is consistent with a previous study (9), and may be due to physiological differences such as lower pH (20). Using samples from all body locations, biological sex could be determined 80.0% of the time, which is greater than expected by chance. However, when biological sex was classified for each body location, the thigh could be correctly classified 100% of the time. One can therefore determine biological sex consistently based on the thigh region, but cannot classify sex with the same certainty using any other skin region. This finding was corroborated by ordinations based on Bray-Curtis distances (Fig. S2), was statistically significant (*F*_1,328_ = 8.0 by PERMANOVA; *P* < 0.001), and was further supported by using indicator species analysis because the thigh region had the most indicator OTUs (Table S2).

10.1128/mSystems.00043-17.2 FIG S2  PCoA ordinations calculated using the Bray-Curtis dissimilarity metric for each of the 10 body locations sampled. The left and right samples from an individual are connected. A single sample per person was collected on body locations (back, navel, torso) where no lines connect the samples. Blue triangles represent samples from males, whereas coral circles represent samples from females. Download FIG S2, PDF file, 0.1 MB. Copyright © 2017 Ross et al. 2017 Ross et al. This content is distributed under the terms of the Creative Commons Attribution 4.0 International license.

10.1128/mSystems.00043-17.7 TABLE S2  Indicator analysis table of biological sex by body location. All *P* values are <0.05. The organism with the highest indicator value per partner was included. Indicator OTUs were defined as having an indicator value threshold of 0.7 and a minimum mean abundance of 10 reads (*P* < 0.05). Download TABLE S2, XLSX file, 0.1 MB. Copyright © 2017 Ross et al. 2017 Ross et al. This content is distributed under the terms of the Creative Commons Attribution 4.0 International license.

Male participants had a significantly larger proportion of sequences affiliated with the *Actinobacteria* phylum (50.8% ± 25.5% versus 38.2% ± 20.9%; *P* < 0.001), whereas female participants had significantly more *Proteobacteria* (18.1% ± 15.9% versus 11.7% ± 17.3%; *P* = 0.001) detected on their skin (Fig. S3). Several OTUs were more abundant on either male or female skin (Table 1). The majority of these bacteria are human skin commensals, whereas *Alloiococcus* in males is associated with the ear canal and associated infections (21, 22). In contrast, women had 11.1-fold more *Lactobacillus*, which is an organism that dominates the vaginal microbiome (23). There were no core OTUs present in one sex that were not core OTUs in the other sex (Table S3).

10.1128/mSystems.00043-17.3 FIG S3  Pie charts illustrating the relative abundance of microbial families present at a relative abundance of >1% and the phyla to which they belong, organized by each of the 10 body locations sampled and by biological sex. Download FIG S3, PDF file, 0.2 MB. Copyright © 2017 Ross et al. 2017 Ross et al. This content is distributed under the terms of the Creative Commons Attribution 4.0 International license.

10.1128/mSystems.00043-17.8 TABLE S3  Rarefied OTU table organized in multiple tabs. The first tab (leftmost tab) contains the original OTU table. The second tab separates the table by individual. The third and fourth tabs separate the OTU table by biological sex. The fifth tab is a condensed table containing only archaeal reads. Sample names are explained in the Table S5 legend. Download TABLE S3, XLSX file, 14.5 MB. Copyright © 2017 Ross et al. 2017 Ross et al. This content is distributed under the terms of the Creative Commons Attribution 4.0 International license.

**TABLE 1:** Microorganisms disproportionately abundant on each biological sex^a^

More abundant on men		More abundant on women	
OTU taxonomic affiliation	Increase in abundance (%)	OTU taxonomic affiliation	Increase in abundance (%)
*Alloiococcus*	344.0	*Lactobacillus*	1,111.4
*Dermabacter*	271.2	*Lactobacillus iners*	440.0
*Brevibacterium*	164.5	*Lautropia*	408.8
*Moraxella*	149.9	*Streptococcus luteciae*	332.7
*Faecalibacterium prausnitzii*	128.7	*Caulobacteraceae*	273.3
*Abiotrophia*	112.4	*Planococcaceae*	273.3
*Neisseria*	109.1	*Sphingomonas*	191.3
*Escherichia coli*	73.1	*Cloacibacterium*	180.9
*Veillonella dispar*	73.0	*Paracoccus*	174.4
*Corynebacterium*	70.5	*Streptococcus*	169.8

a OTUs that were present on a minimum of 30% of samples of that sex are shown.

### Cohabiting partners can be predicted on the basis of skin microbiome profiles.

**FIG 5:**
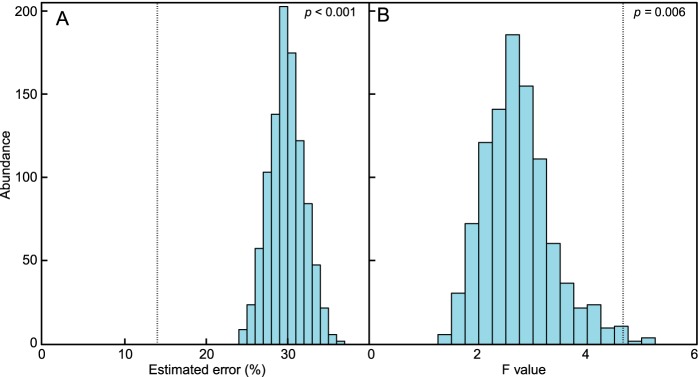
Bar plots of the data set with the correct couple composition compared to randomly assorted incorrect pairings. Distribution of the estimated supervised learning error rates (A) and PERMANOVA *F* values of 1,000 unique artificially shuffled partner pairings (B). The dotted lines represent the position of the result from the correctly matched couples’ data set (*P* < 0.001 and *P* = 0.006).

By analyzing all samples together, random forest modeling found that couples’ samples could be matched correctly 86% ± 6.1% of the time, which is 6.5-fold greater than chance and always had a lower error rate than the 1,000 randomized groupings of samples from couple participants (*P*< 0.001; Fig. 5A). Couple H05 had distinct microbial communities with more indicator OTUs than the other couples (Table S4). When the 34 samples from couple H05 were removed from the random forest modeling, there was no significant difference in the model’s ability to classify the remaining nine couples (*P* = 0.68). This result indicates that an outlier did not significantly influence the analysis. When the same 1,000 randomized groupings of samples from all couple participants were analyzed using PERMANOVA, the incorrect partner *F* values were significantly lower than the correct couple pairs (*P* = 0.006; Fig. 5B). When the PERMANOVA analysis was divided by body location, partners possessed the most similar microbial communities on their feet, eyelids, and back compared to incorrect couple pairings (Fig. S4).

10.1128/mSystems.00043-17.4 FIG S4  Bar plots of the distribution of the PERMANOVA *F* values of 1,000 unique artificially shuffled partner pairings for each body location (A to J). The dotted line represents the position of the result from the correctly matched couples’ data set. Download FIG S4, PDF file, 0.04 MB. Copyright © 2017 Ross et al. 2017 Ross et al. This content is distributed under the terms of the Creative Commons Attribution 4.0 International license.

10.1128/mSystems.00043-17.9 TABLE S4  Indicator analysis table of couples by body location. The organism with the highest indicator value per partner was included. For each body location, only partners that had at least one indicator were listed. An indicator was defined as having an indicator value threshold of 0.7 and a minimum mean abundance of 10 reads (*P* < 0.05). Download TABLE S4, XLSX file, 0.1 MB. Copyright © 2017 Ross et al. 2017 Ross et al. This content is distributed under the terms of the Creative Commons Attribution 4.0 International license.

To further assess the similarity between couples’ samples, a Bray-Curtis distance matrix was created to determine the number of times the sample with the lowest distance, and therefore the most similar microbial community, belonged to the same individual, their partner, or another participant (Fig. 3). When only the closest nonself samples were tested, several body locations had microbial communities that were closest to their partner. For example, 35% of couples’ foot samples were closest to those of their partner, which is ~7-fold higher than expected by chance (Fig. 3B). The torso (21%), navel (20%), and eyelid (17.5%) were also closer to their partner more frequently than expected by chance.

Although the closest nonself samples matched their partner more often than expected by chance, the majority of the samples matched one of the other 18 participants in the study. We hypothesize that this may be partially attributed to shared factors between these participants, such as biological sex. Indeed, the majority of nonself samples that did not match to a partner matched with a participant of the same sex (62.4%; *P* = 0.005). Partners can therefore be correctly paired by random forest modeling better than using the closest matching sample method in part because random forest modeling is restricted to couples of different sexes. The closest nonself, opposite-sex matches were also analyzed to correct for the influence of biological sex. Although there was little difference in the majority of body locations, the thigh and torso samples matched partners correctly more frequently only when considering opposite-sex individuals (Fig. 3C).

Investigating associations of samples with other samples from self, partners, and others was also performed at a 99% sequence identity threshold to determine whether a data set with higher resolution was better able to match self or partners. The 99% identity data set showed a significant improvement in linking samples to other samples from the same individual using Bray-Curtis distances (78% versus 69%; *P* = 0.003) but only modestly increased average partner identification, albeit insignificantly (23.1% versus 20.3%; *P* = 0.09). This result was corroborated by random forest modeling. The models were worse at classifying partners (80% ± 5% versus the original 86% ± 6%; Fig. S5), which was partially due to an increased inability to classify couple 10 (data not shown), who had been living together for the least amount of time compared to the other participants. However, although random forest modeling was worse at classifying the true partners, it was also worse at classifying the incorrectly paired partners (Fig. S5), indicating no difference in overall classification ability.

10.1128/mSystems.00043-17.5 FIG S5  Bar plots of the data set with the correct couple composition compared to randomly assorted incorrect pairings. Distribution of the estimated supervised learning error rates at 97% sequence identity (A) and 99% sequence identity (B) of 1,000 unique artificially shuffled partner pairings. The dotted line represents the position of the result from the correctly matched couples’ data set (*P* < 0.001 for both analyses). Download FIG S5, PDF file, 0.03 MB. Copyright © 2017 Ross et al. 2017 Ross et al. This content is distributed under the terms of the Creative Commons Attribution 4.0 International license.

An indicator species analysis was also performed to characterize couples based on highly abundant organisms that were unique to them (Table S4). Only 3 of the 10 couples had at least one indicator OTU. One of these couples had seven indicator species. This couple had several unique factors that were not observed in any of the other participants. The couple reported more time outdoors, a higher exercise frequency, flaky skin, and a different racial heritage than the other participants. Future skin microbiome studies would need to be conducted to assess the effects of race, exercise frequency, and time spent outdoors. The couple did not have a clinical diagnosis of any skin ailments that may cause flaky skin, such as psoriasis. Specific body locations had a higher number of indicators per couple. Additionally, analyzing indicators by each individual body location resulted in a higher number of indicators for each couple.

We hypothesize that the mode of transmission of the microbiota between partners is through a combination of the built environment and direct contact. Humans shed over one million biological particles per hour (24), changing the composition of the surfaces they touch and the rooms they occupy (25, 26). Indeed, microbiome individuality enabled hand microbial communities to be linked to computer keyboards based on physical contact (27). Forensic studies have shown that humans can be correctly matched, based on microbiome profiles, to the fabrics they grasp (28) and personal objects, such as cell phones and shoes (29). It is therefore plausible that partner shedding and direct contact affects the other inhabitants of an individual’s primary residence. Sharing of microbiota was most apparent by sampling feet, which can be explained by feet being in direct contact with home surfaces. Indeed, homes have been shown to have microbiota that are signatures of their inhabitants (14, 15). Daily direct contact between cohabiting individuals would further increase microbiota sharing.

Future skin microbiome studies should include same-sex couples to answer intriguing questions about how intimately living with a member of the same sex affects the microbiome. For example, skin regions that are heavily influenced by biological sex, such as the thigh (Fig. S2), or hands (9), may be more effective at classifying same-sex couples because biological sex would no longer be a confounding factor. We predict that homosexual couples would be matched more successfully than heterosexual couples because they would share more similar skin microbiota both from sharing a location and biological sex.

### Hygiene, pets, and allergies correlate with the microbiota of body sites.

Lifestyle choices for each participant, such as time spent outdoors, pet ownership, and alcohol consumption were analyzed to determine whether these factors exhibited any significant effect on skin microbiota (Table S5). Although the sample size was small (17 body locations; 20 participants), several factors were nonetheless significant after using Bonferroni’s correction (Table S1). The following lifestyle choices may warrant future experimentation with a larger sample size to further elucidate their impact on skin communities.

10.1128/mSystems.00043-17.10 TABLE S5  Metadata table containing all participant survey responses, PCR setup information, and sample codes. Sample identification (ID) names are coded as follows. H01 to H10 signify each couple, while A and B differentiate individuals within a couple. The numbers in the sample ID names indicate the sample sites as follows: 01, left upper eyelid; 02, right upper eyelid; 03, left outer nose; 04, right outer nose; 05, left inner nostril; 06, right inner nostril; 07, left armpit; 08, right armpit; 09, torso; 10, back; 11, navel; 12, left inner thigh; 13, right inner thigh; 14, bottom of left foot; 15, bottom of right foot; 16, left palm of hand; 17, right palm of hand. Download TABLE S5, XLSX file, 0.1 MB. Copyright © 2017 Ross et al. 2017 Ross et al. This content is distributed under the terms of the Creative Commons Attribution 4.0 International license.

Participants who consumed multiple servings of alcohol per day had significantly less-diverse communities, based on the Shannon index, than those who consumed one serving per month (Table S1). This result was corroborated by PERMANOVA data, which identified alcohol consumption as a metadata category contributing to explained variation (Fig. 4). An indicator of the highest consumption rate of alcohol was *Brevibacterium* (indicator value of 0.70; mean of 129 versus a mean of 2 in the lower consumption groups). This organism possesses an alcohol dehydrogenase (30), and given that ethanol is secreted through the sweat glands (31), it is possible that higher rates of alcohol consumption could feasibly impact members of the skin microbiota. However, it should be noted that many organisms possess this enzyme, and additional studies are needed to properly determine the effect alcohol has on skin microbiota.

Spending more time outdoors and owning pets were associated with higher levels of microbial skin community diversity (Table S1). On the basis of Shannon indices, participants who spent more than 4 h per day outside had more-diverse communities than people who were outside for less than an hour per day. Pet ownership also had a significant effect (increase) on the measured microbial diversity of thigh and nostril skin samples, which is in accordance with previous research that demonstrated that homes with dogs had higher levels of diversity (14, 32). These studies have stated that diversity is increased both by dog-associated taxa and their need to be outdoors daily. The nostrils are hypothesized to have experienced a shift in microbiota due to inhaling biological particles shed from the animals.

Use of a large number of skin products was correlated with higher diversity, but this may be linked to other sex-associated factors. In particular, facial regions such as the eyelids had higher diversity for participants who applied facial cleansers, moisturizers, or cosmetics, which were predominantly female participants. Body regions whose microbiota varied by biological sex, such as the thighs due to the influence from the vaginal microbiome, therefore also appeared to be strongly correlated with sex-specific hygiene products, such as facial cleansers, although these products would not have an effect on the microbial community of body regions they do not directly contact. A study that mapped the 3D molecular cartography of human skin noted that skin composition was influenced by hygiene product application (1). Our study further indicates that this lasting impact on the skin environment may increase the diversity level of facial skin.

The inner nostril was included as a skin region because it contains sebaceous and sweat glands, as well as a keratinized epidermal layer that is more similar to skin than the nasal cavity (33). Participants with higher numbers of allergies had significantly more-diverse nostril and outer nose communities (Table S5). Individuals with dairy, wheat, and strawberry allergies exhibited higher diversity, whereas those with dust allergies had lower diversity. Asthma sufferers have been reported to have higher microbial diversity of the lung microbiome (34–36). It is therefore possible that increased diversity at the entrance to the respiratory tract may influence other ailments, such as allergies. Allergies did not appear to have an effect on any other body locations.

### Study limitations.

A limitation of the study was a limited sample size of 20 individuals. The goal of the current study was to sample from a wide range of body locations in order to identify the regions that are most useful for linking couples via their skin microbial profiles. This was conducted instead of sampling a low number of sites from many individuals to avoid missing key trends in the microbiota across the body. For example, if we had only sampled the thighs of many couples, we would not have been able to elucidate any cohabitation effect on the skin microbiome. Now that trends from each body location have been observed, future studies can focus on sampling a broader range of participants while reducing the number of samples required per participant, especially because individuality was pronounced.

Additionally, this study sequenced the V3-V4 region of the 16S rRNA gene. Research in this area has been progressing rapidly and has resulted in numerous research articles and reviews discussing the inherent biases in each primer region (37–40). It is possible that relative abundances of certain organisms, such as *Staphylococcus* and *Propionibacterium*, would be altered if a different region had been used for this study, such as the V1-V3 region (39). Indeed, mismatches of only a few base pairs can result in poor amplification of taxa that are abundant on skin, as was the case with *Propionibacterium* in a previous study (41). It has therefore been suggested by Zeeuwen and colleagues that a skin microbiome-specific primer set should be designed that is optimized for bacteria found on human skin, which does not necessarily need to exclude the V4 primer region (42). Further validation would include sequencing a defined mock community with staggered concentrations to monitor any potential biases that may occur within the selected primer region or two-step amplification protocol.

There was also limited ethnic diversity; nearly all participants were of Caucasian heritage. The cohabiting couple of non-Caucasian descent had more indicators than the other couples. It is unknown the degree to which racial heritage attributed to this change in microbial diversity compared to other factors, such as reported skin conditions, time spent outdoors, and activity levels. Additional studies will include a higher diversity of participants to determine the effects that racial heritage and related social changes may have on the skin microbiome. Indeed, other microbiome studies have concluded that race was a significant factor governing the microbiome of the vagina (23, 43) and gut (44).

### Conclusion.

This study was the first to analyze the distribution of microorganisms on cohabiting couples by sampling a wide range of skin regions. Although body location and individuality had the most substantial influences on the skin microbiome of sampled cohabiting couples, machine learning approaches were nonetheless able to classify samples from cohabiting couples in >86% of test cases. Couples were most similar based on foot microbiota, likely reflecting the collection and distribution of dust from floors to all occupants of a home. In contrast, the inner thigh region was the best indicator of individuality and biological sex. Possessing pets, consuming smaller quantities of alcohol, and exercising more were associated with higher levels of microbial diversity.

## MATERIALS AND METHODS

### Ethics.

This study has been approved by the Office of Research Ethics at the University of Waterloo (ORE no. 20993). The following procedures were conducted in accordance with the approved documentation. Written consent was received from all individuals and cannot be linked to completed surveys or samples. All participants remain anonymous.

### Sample collection.

A total of 17 body regions of 10 cohabiting and sexually active couples living in southwestern Ontario in Canada were sampled to determine the distribution of their microbial communities (Fig. 1). All participants reported being healthy heterosexual adults between the ages of 20 to 49 years and had lived together for periods ranging from 4 months to 14 years. For consistency, the study included mostly Caucasian subjects (18 subjects), although one East Asian participant and one Asian participant were also included. The upper eyelids, outer nostrils, inner nostrils, armpits, torso, back, navel, inner thighs, bottom of feet, and palms of hands were sampled by the participants themselves using sterile foam swabs. While applying moderate pressure, skin was swabbed five times in a forwards and backwards motion. The swab was then rotated and repeated in adjacent areas at the same body site for a total of 20 strokes per swab. Sample swabs were returned to their initial plastic storage container and frozen at −20°C until DNA extraction. All participants provided comprehensive metadata for analysis (see Table S5 in the supplemental material). These data were collected to test whether lifestyle choices also affected the skin microbiome and to determine whether any of the participants had any confounding factors that may impact the results. Although all categories were analyzed to determine whether any lifestyle choices had an effect, only significant factors are discussed.

### DNA extraction and amplification.

Genomic DNA was extracted using the PowerSoil-htp 96-well DNA isolation kit (Mo Bio Laboratories) according to a previously published protocol (45). The beadbeating manufacturer’s protocol was used at the speed setting of 20 for 10 min on a Mixer Mill MM400 plate shaker (Retsch) with a plate adapter set (Mo Bio Laboratories). A final 75-μl volume of DNA was eluted and stored at −20°C in the EDTA-free elution solution supplied in the kit.

A 464-bp fragment of the V3-V4 region of the 16S rRNA gene was amplified by using the universal prokaryotic primers Pro341Fi (5′-CCTACGGGNBGCASCAG-3′) and Pro805Ri (5′-GACTACNVGGGTATCTAATCC-3′) (46). The V3-V4 region has been used in multiple skin microbiome studies (25, 47–49) and has been experimentally shown to produce the closest sequence agreement to known low-diversity mock community samples (40). However, previous studies have commented on the potential for bias between this region and the V1-V3 region, particularly with *Propionibacterium* and *Staphylococcus* (37–39). The primers were modified to include adapters for binding the flow cell (Illumina), a 6-base barcode for multiplexing, and complementary forward and reverse regions required for Illumina primers, as described previously (50). Two rounds of PCR were conducted targeting the same 16S rRNA gene region. The first round was conducted for 25 cycles without the Illumina adapters, followed by a second round of 15 cycles in order to attach the adapters. We have found that this procedure improves yield and specificity for low-biomass samples from a wide range of skin and surface swab DNA extracts. Two rounds of amplification did not result in a difference in community composition according to a preliminary subset of four samples that were sequenced using both protocols with one round and two rounds of amplification (data not shown). The first PCR amplification mix contained 2.5 μl of 10× ThermoPol *Taq* buffer (New England Biolabs), 1.5 μl of 10 mg ml^−1^ bovine serum albumin (BSA), 0.05 μl of 100 mM total deoxynucleoside triphosphates (dNTPs) (New England Biolabs), 0.05 μl of each 100 μM forward and reverse primer (Integrated DNA Technologies), 0.125 μl of 5 U μl^−1 ^*Taq* polymerase (New England Biolabs), 1 to 10 ng of template DNA (3 μl for the majority of samples), and nuclease-free PCR-grade H_2_O to a 25-μl total reaction volume. The reaction was run on either the T100 thermal cycler (Bio-Rad) or the C1000 thermal cycler (Bio-Rad) at 95°C for 30 s (initial denaturation) and 25 cycles, with 1 cycle consisting of 95°C for 15 s (denaturation), 55°C for 30 s (annealing), 68°C for 60 s (extension), and a final extension of 68°C for 10 min.

The second PCR amplification mix contained 2.5 μl of 10× ThermoPol *Taq* buffer, 1.5 μl of 10 mg ml^−1^ BSA, 0.05 μl of 100 mM total dNTPs, 0.05 μl of 100 μM forward primer, 1 μl of 5 μM reverse primer, 0.125 μl of 5 U μl^−1 ^*Taq* polymerase, 1 μl of product from the first PCR, and 18.8 μl of nuclease-free PCR-grade H_2_O to a total reaction mixture volume of 25 μl. The reaction was run on the T100 thermal cycler or the CFX96 real-time system at 95°C for 30 s and 15 cycles, with 1 cycle consisting of 95°C for 15 s, 55°C for 30 s, 68°C for 60 s, and a final extension of 68°C for 10 min. One sterile swab control per kit and one negative template control per 96-well plate were included to monitor potential contamination. Each reaction was performed in triplicate to eliminate any potential PCR bias (51). All PCR amplifications were prepared in a PCR hood that was UV treated for 15 min. All triplicate PCR amplifications were pooled and stored at −20°C until further use.

### Illumina library preparation.

Pooled samples were quantified using the AlphaView band analysis tool (ProteinSimple). Relative concentrations of each product were determined, and each 96-well plate was pooled using an equal quantity of PCR product. The pools were purified using the manufacturer’s protocol for the Wizard SV gel and PCR cleanup system (Promega) and stored in nuclease-free H_2_O at −20°C until library quantification.

The purified pools were quantified using the Qubit 2.0 fluorometer (Life Technologies) with the Qubit dsDNA (double-stranded DNA) HS (high-sensitivity) assay kit (Invitrogen) and diluted to 6 nM. The 6 nM pools were merged into a single 100-μl pool, ensuring that each sample had an equivalent amount of PCR product in the final pool. The correct DNA concentration was determined using Qubit, the qPCR (quantitative PCR) PerfeCTa NGS (next-generation sequencing) library quantification kit for Illumina sequencing platforms (Quanta Biosciences), and gel quantification. The concentrations calculated from all quantification methods were compared to ensure consistent concentration readings and were subsequently stored at −20°C until sequencing occurred. The library was quantified the day of sequencing using a Qubit fluorometer to ensure that freezing had not altered the DNA concentration.

### Illumina sequencing.

High-throughput sequencing on a MiSeq instrument (Illumina) was used to analyze the amplified V3-V4 region of the 16S rRNA gene. The MiSeq reagent v2 kit-500 cycles (Illumina) was used according to the manufacturer’s protocol to prepare the final quantified pool for sequencing. A 10% PhiX control was included to increase sample diversity. In brief, the library and the PhiX control were denatured with 0.2 N NaOH and diluted to 8 pM, before being merged 10:1. The pooled library/PhiX solution was loaded onto the MiSeq reagent v2 cartridge (Illumina) and sequenced.

### Assembly of sequence data.

The following bioinformatic pipeline was composed entirely of open-source software and was managed by AXIOME (*a*utomation, e*x*tension, and *i*ntegration *o*f *m*icrobial *e*cology) version 1.5 (52). The generated paired-end sequences were assembled with PANDAseq version 2.8 (53) using the default parameters of a quality threshold of 0.9, a minimum sequence overlap of 1 bp, and a minimum read length of 32 bp. The assembled sequences were analyzed by QIIME (*q*uantitative *i*nsights *i*nto *m*icrobial *e*cology) version 1.9.0 (54). The sequences were then clustered *de novo* with UPARSE (55) at 97% and 99% sequence identity, which also removed chimeras and sequences that appeared only once in the data set (55). The clustered sequences were then aligned with PyNAST version 1.2.2 (56). RDP version 8.1 (57) was used to assign all taxonomy with a minimum confidence of 80%, using the most recent Greengenes database version 13.8 (58).

Samples with fewer than 5,293 reads were removed. These samples did not have a visible band on the agarose gel but had been included in case there was sufficient DNA for downstream analyses. All of the no-template controls (NTCs) and sterile swab controls contained less than half the number of reads as this cutoff. This resulted in the removal of the following 10 samples in the human data set: three armpit and foot samples, as well as one sample each from back, thigh, hand, and torso. Rarefication was used to control for uneven sequencing depth by equalizing the number of reads per sample (Table S3).

### Statistical analyses.

All analyses were generated using a Bray-Curtis distance matrix when applicable. Sample alpha diversity was measured using the Shannon index and the total number of observed OTUs. Nonparametric two-sample *t* tests were calculated to determine the significance of each of the 79 metadata categories (Table S5) using the multiple_rarefaction.py, alpha_diversity.py, collate_alpha.py, and compare_alpha_diversity.py commands in QIIME. Multiple rarefactions were conducted with a minimum size of 100 reads and a maximum size of 5,290 reads, increasing in intervals of 100 sequences after creating 10 rarefactions for a total of 530 rarefactions. Bonferroni’s correction was used to avoid false significance from a data set of 330 samples.

Sample beta diversity was measured using ordinations and permutational analysis of variance (PERMANOVA). Principal-coordinate analysis (PCoA) ordinations were used to visualize the microbial variation between the samples. Ordinations were created with the phyloseq (version 1.14.0) and ggplot2 (version 2.1.0) packages in RStudio (59). PERMANOVA calculates the percent variation explained by each metadata category and was run using the adonis function from the vegan package (version 2.4-0) in R with 1,000 permutations.

Indicator species were classified to determine which organisms were highly abundant across various metadata categories. Core species, classified as OTUs that have at least one sequence per sample in a particular category, were also determined to identify organisms that were ubiquitous on all skin samples. The Dufrene-Legendre indicator species analysis was conducted to determine whether specific OTUs were indicative of a specific metadata category (60). An indicator was defined as having a significant *P* value (<0.05) and an indicator value threshold of 0.7 with a minimum mean abundance of 10 reads.

Random forest modeling was used to determine the accuracy with which samples can be correctly classified to couples. The supervised_learning.py command in QIIME was used with the options of creating 1,000 trees and 10-fold cross validation. All possible incorrect couple pairings, where all couples were matched with a nonpartner participant of the opposite sex, were generated using the module itertools.permutations as a custom script in Python.

### Data availability.

The sequence data associated with this article are available in the Sequence Read Archive (SRA) under BioProject accession number PRJNA345497.
