# Supplementary figures and images for: The Skin Microbiome of Cohabiting Couples
Source: mSystems. 2017 Jul 20;2(4):e00043-17. doi: 10.1128/mSystems.00043-17 (PMC5527301; doi:10.1128/mSystems.00043-17)

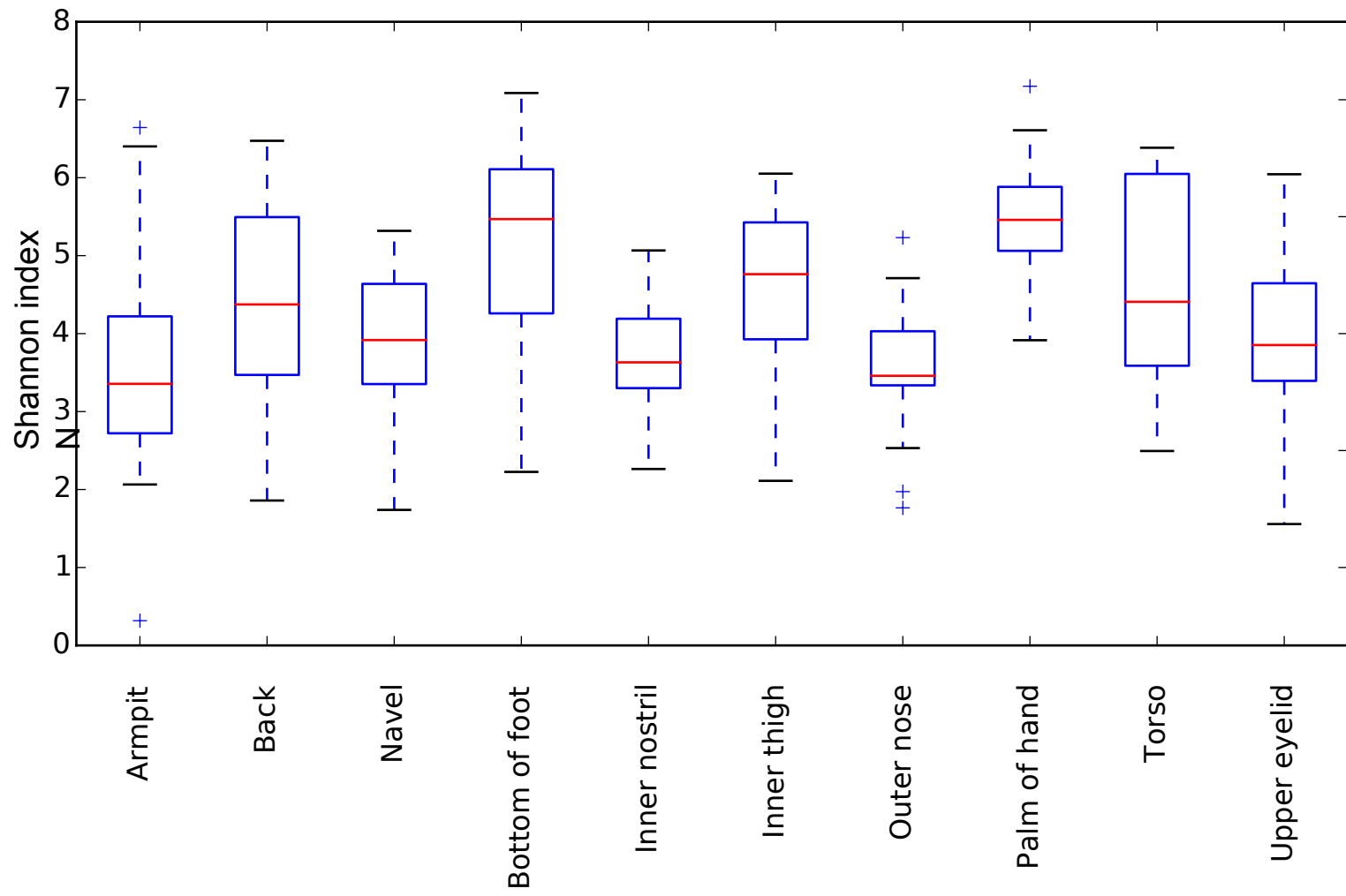

Supplement: FIG S1 [file sys004172121sf1.pdf]

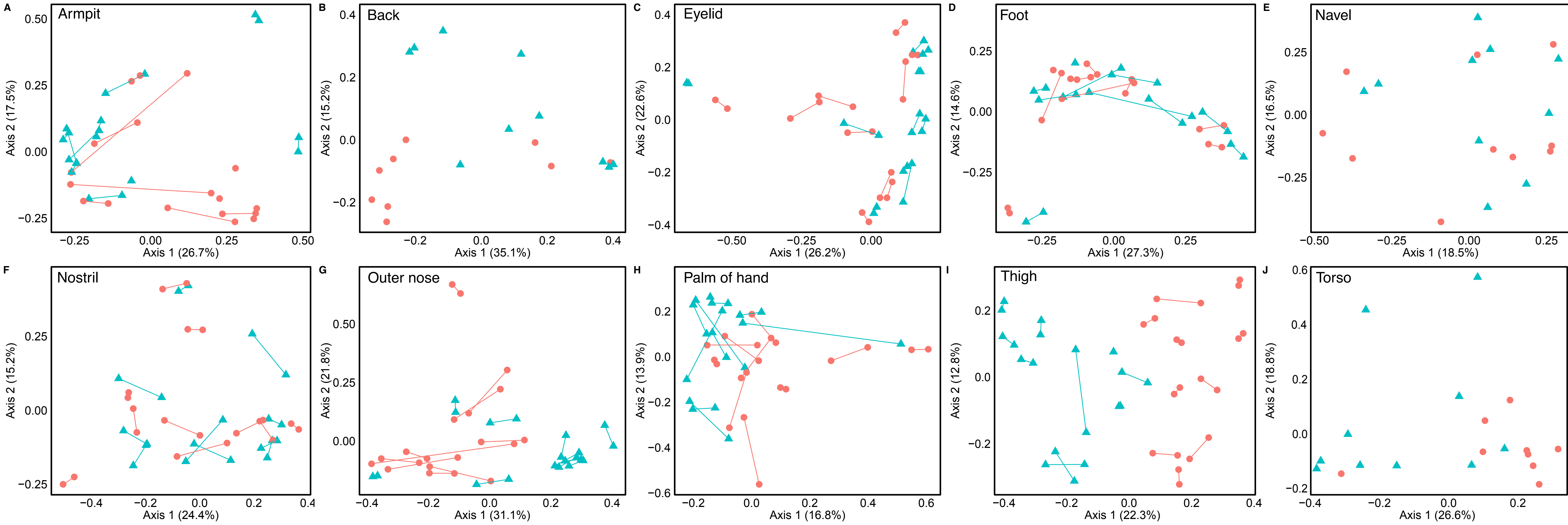

Supplement: FIG S2 [file sys004172121sf2.pdf]

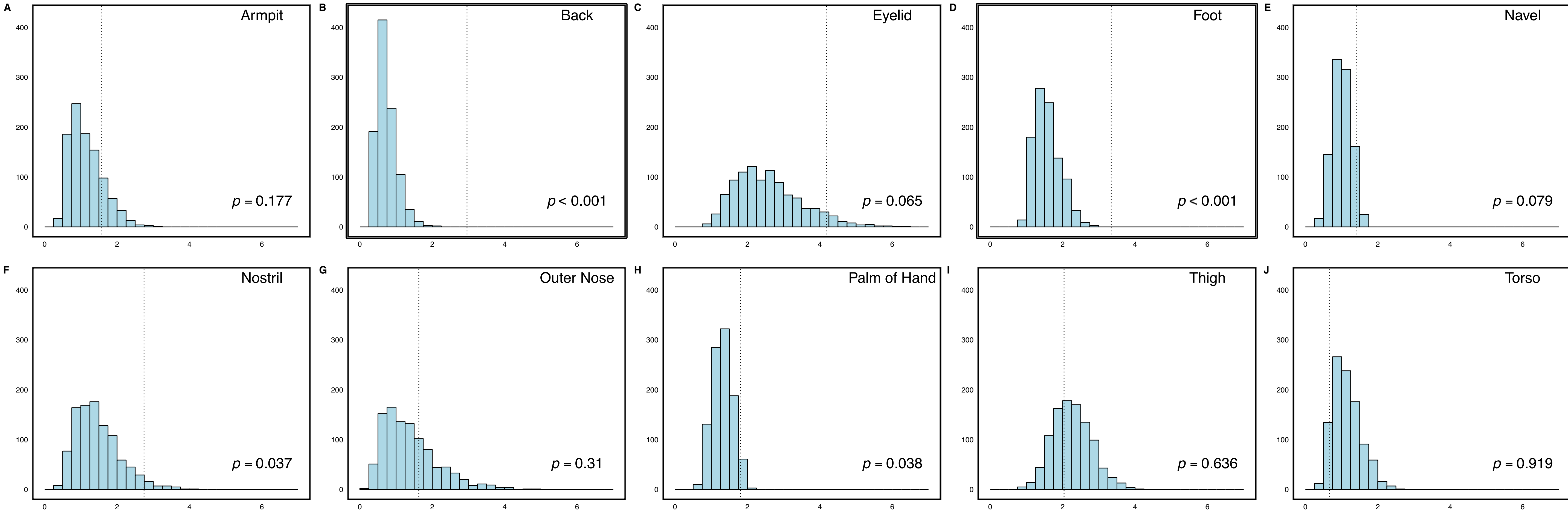

Supplement: FIG S4 [file sys004172121sf4.pdf]

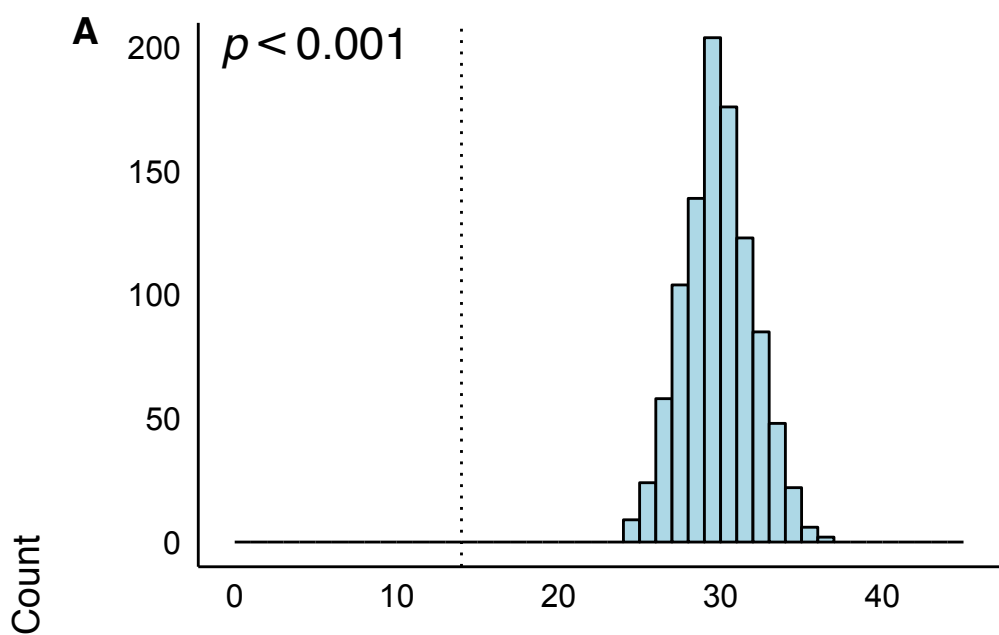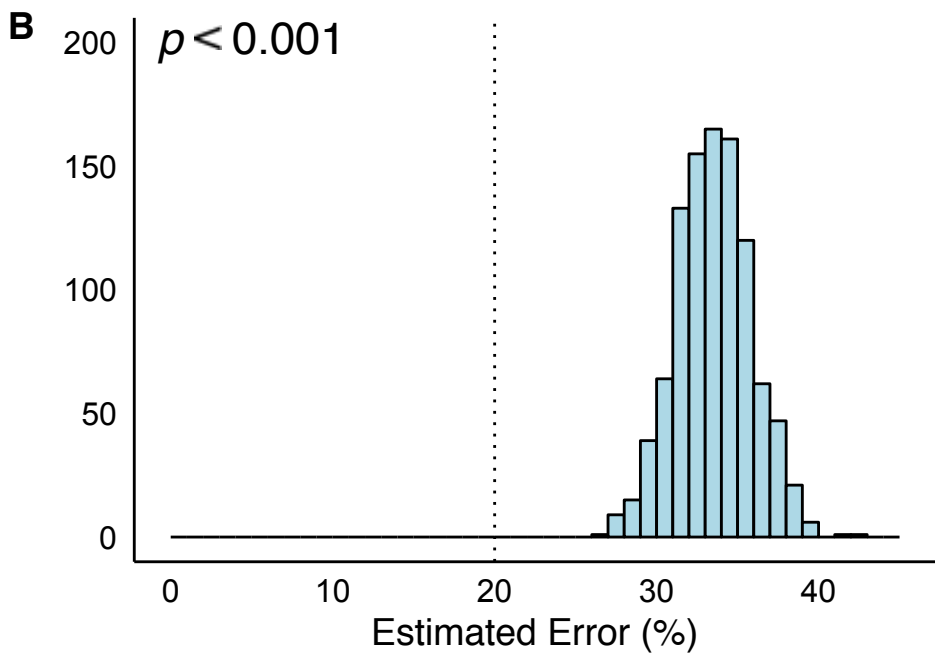

Supplement: FIG S5 [file sys004172121sf5.pdf]
