# Supplementary material for: The Skin Microbiome of Cohabiting Couples
Source: mSystems. 2017 Jul 20;2(4):e00043-17. doi: 10.1128/mSystems.00043-17 (PMC5527301; doi:10.1128/mSystems.00043-17)
Supplement: FIG S3 [file sys004172121sf3.pdf]

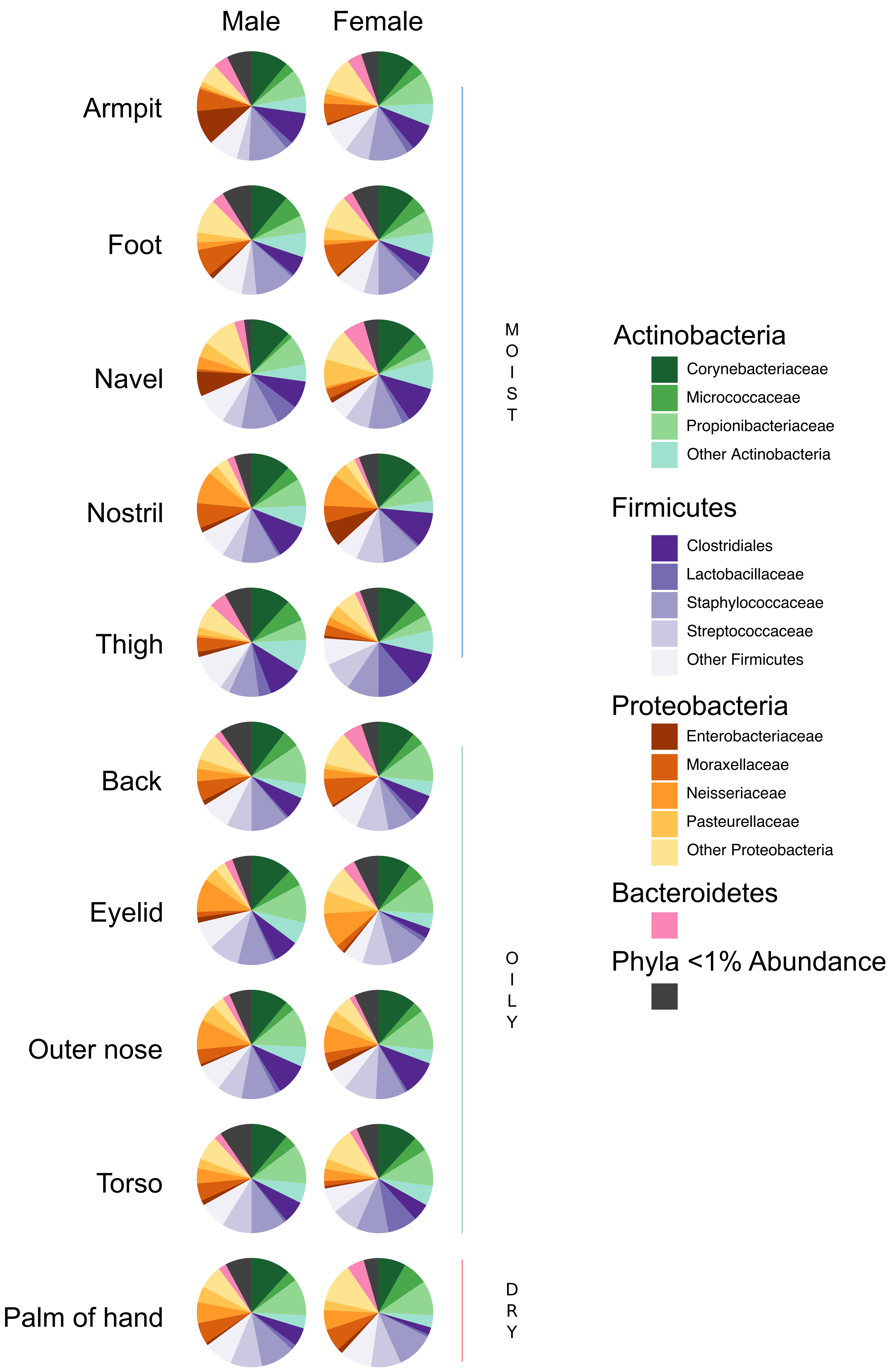
Male
Female
Armpit
Foot
Navel
Nostril
Thigh
Back
Eyelid
Outer nose
Torso
Palm of hand
MOIST
OILY
DRY
Actinobacteria
Corynebacteriaceae
Micrococcaceae
Propionibacteriaceae
Other Actinobacteria
Firmicutes
Clostridiales
Lactobacillaceae
Staphylococcaceae
Streptococcaceae
Other Firmicutes
Proteobacteria
Enterobacteriaceae
Moraxellaceae
Neisseriaceae
Pasteurellaceae
Other Proteobacteria
Bacteroidetes
Phyla <1% Abundance
